# News Media Coverage of the Problem of Purchasing Fake Prescription Medicines on the Internet: Thematic Analysis

**DOI:** 10.2196/45147

**Published:** 2023-03-21

**Authors:** Hamzeh Almomani, Nilesh Patel, Parastou Donyai

**Affiliations:** 1 School of Pharmacy University of Reading Reading United Kingdom; 2 Department of Pharmacy and Forensic Science King’s College London London United Kingdom

**Keywords:** prescription medicine, internet, online pharmacy, fake medicine, media, newspaper article, Theory of Planned Behavior, thematic analysis

## Abstract

**Background:**

More people are turning to internet pharmacies to purchase their prescription medicines. This kind of purchase is associated with serious risks, including the risk of buying fake medicines, which are widely available on the internet. This underresearched issue has been highlighted by many newspaper articles in the past few years. Newspapers can play an important role in shaping public perceptions of the risks associated with purchasing prescription medicines on the internet. Thus, it is important to understand how the news media present this issue.

**Objective:**

This study aimed to explore newspaper coverage of the problem of purchasing fake prescription medicines on the internet.

**Methods:**

Newspaper articles were retrieved from the ProQuest electronic database using search terms related to the topic of buying fake prescription medicines on the internet. The search was limited to articles published between April 2019 and March 2022 to retrieve relevant articles in this fast-developing field. Articles were included if they were published in English and focused on prescription medicines. Thematic analysis was employed to analyze the articles, and the Theory of Planned Behavior framework was used as a conceptual lens to develop the coding of themes.

**Results:**

A total of 106 articles were included and analyzed using thematic analysis. We identified 4 superordinate themes that represent newspaper coverage of the topic of buying prescription medicines on the internet. These themes are (1) the risks of purchasing medicines on the internet (eg, health risks and product quality concerns, financial risks, lack of accountability, risk of purchasing stolen medicines), (2) benefits that entice consumers to make the purchase (eg, convenience and quick purchase, lower cost, privacy of the purchase), (3) social influencing factors of the purchase (influencers, health care providers), and (4) facilitators of the purchase (eg, medicines shortages, pandemic disease such as COVID-19, social media, search engines, accessibility, low risk perception).

**Conclusions:**

This theory-based study explored the news media coverage of the problem of fake prescription medicines being purchased on the internet by highlighting the complexity of personal beliefs and the range of external circumstances that could influence people to make these purchases. Further research is needed in this area to identify the factors that lead people to buy prescription medicines on the internet. Identifying these factors could enable the development of interventions to dissuade people from purchasing medicines from unsafe sources on the internet, thus protecting consumers from unsafe or illegal medicines.

## Introduction

### Background

People are increasingly turning to the internet for many of their needs, and it is anticipated that the number of people obtaining their medicines via the internet will also increase significantly in the near future [1]. This shift is attributed to the advantages that the internet marketplace of medicines offers for consumers, such as convenience, privacy, and accessibility 24 hours a day and 7 days a week [2]. It contrasts with traditional “brick and mortar” pharmacies, which are associated with longer queues, less privacy, and shorter opening times. Additionally, the COVID-19 pandemic has accelerated the purchase of medicines via the internet [3]. A study conducted by researchers from Hungary found that the rate of medicines purchased on the internet increased from 4.2% in 2018 to 44.2% in March 2020 [3].

Many illegal sellers of medicines operate on the internet and fail to meet national or international pharmacy regulations [4]. These sellers offer a wide range of medicines, including prescription medicines, without a prescription being issued and without medical supervision. According to an estimate, 95% of pharmacies on the internet are operating illegally [5]. Several researchers have explored the internet availability of prescription medicines offered by illegal sellers and the accessibility of those medicines without the need for prescriptions. Researchers from the United States conducted an internet-based study to check the availability of prescription psychiatric medicines using Google [6]. They found that 147 (88%) of 167 internet-based pharmacies offered psychiatric drugs without a need for a prescription. In another study, the availability of antibiotics was explored using Yahoo and Google search engines [7]. A total of 20 unique URL addresses were analyzed. The researchers found that 16 (80%) of 20 websites offered antibiotics without a need for a prescription.

The internet availability and accessibility of prescription medicines in the absence of medical oversight present many patient safety risks. Risks include the possibility of misusing medicines and consuming contraindicated medicines [5]. One of the most serious risks associated with purchasing medicines on the internet relates to purchasing and consuming fake medicines (ie, falsified medicines).

Fake, falsified, or counterfeit medicines are terms that have been used interchangeably in many studies. All these terms refer to medicines produced under illegal and unregulated conditions [8]. However, some health organizations differentiate between these terms. Counterfeit medicine “are medicines that do not comply with intellectual property rights or that infringe trademark law,” while falsified medicines are “fake medicines that are passed off as real medicines” [9]. In other words, counterfeit medicines are linked more to the infringement of intellectual properties, while the term falsified describes medicines that threaten public health. The term “fake medicines” has been deemed to be the best term for communicating with the public about falsified medicines [10]. Our study focuses on falsified medicines, with the term “fake medicines” used to represent these kinds of medicines.

Illegal sellers of medicines on the internet are a potential source of fake medicines, and according to the World Health Organization (WHO), 1 of 2 medicines sold on the internet is fake [11]. A report published by the UK government in July 2022 confirmed that over 285,000 fake medical products were seized across the United Kingdom by Interpol [12]. Thus, people could put themselves at serious risk if they purchase and consume medicines from the internet.

To combat the internet sale of fake medicines, Interpol established Operation Pangea in 2008, which targeted the illegal sellers of medicines (ie, the supply side) [13]. This operation has resulted in the detection and confiscation of millions of fake medicines, as well as many illegal sellers’ websites being closed. On the demand side (ie, people who purchase medicines from the internet), several public awareness campaigns have been run by different national and international organizations to alert consumers about the risks of fake medicines available on the internet (eg, Alliance for Safe Online Pharmacies campaigns, #FAKEMEDS, and Fight the Fake campaigns). Besides warning consumers about the risks of fake medicines available on the internet, these campaigns aim to educate them on how to purchase medicines on the internet safely. Despite the abundance of those campaigns, some people continue to buy their prescription medicines over the internet without input from a health care professional. For example, in the United Kingdom, which has a well-established health care system, it was estimated that 1 in 10 people had bought a fake medical product from illegal sellers via the internet in 2020 [12]. This underresearched issue has been highlighted in newspaper articles in the past few years, particularly during the COVID-19 pandemic, when more people turned to the internet to obtain various products [14]. Newspaper coverage of this problem can act in 2 ways. First, taken at face value, this coverage can provide accurate information, for example, on the views and experiences of public and health professionals, including patients, experts, and special authorities that fight fake medicines bought through internet sources (eg, Interpol, WHO, and pharmaceutical companies). There is precedence for using newspapers as a source of data in research [15-18]. Second, newspaper coverage can have a direct effect on what people think by sharping the news agenda and influencing peoples’ views. Thus, newspapers can influence people based on what they emphasize or deemphasize through their content and how this content is curated and presented [19-21]. In the context of this study, newspaper stories could potentially help influence social norms.

Accordingly, this study aims to explore the newspaper coverage of the problem of purchasing fake prescription medicines on the internet using the Theory of Planned Behavior (TPB) as a conceptual lens to develop a coding of themes to encapsulate how this issue is presented through news stories.

### About the TBP

**Figure 1:**
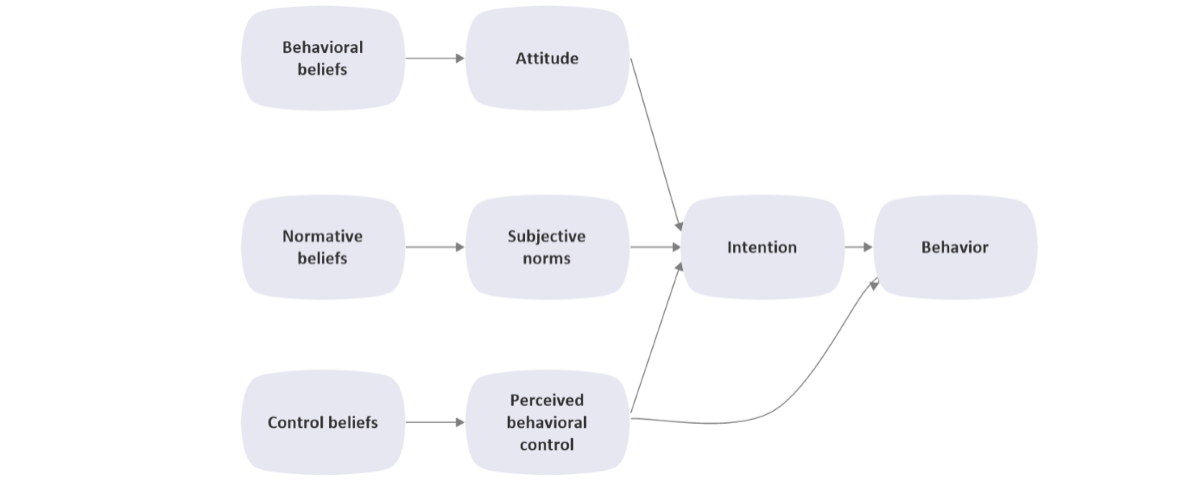
The Theory of Planned Behaviour (TPB) framework.

The TPB is a behavioral theory introduced by Ajzen in 1991 (Figure 1). The logic for using it in this study is that it can be used to interpret peoples’ behaviors based on their intentions [22].

This theory stipulates that peoples’ actual behavior is determined by their intentions, which in turn, are predicted by their attitude toward the behavior, subjective norms, and perceived behavioral control [22]. Each of these 3 direct predictors of intention is influenced by indirect determinants, which are a set of salient beliefs, namely, behavioral beliefs, normative beliefs, and control beliefs. Behavioral beliefs about the consequences of a behavior (ie, beliefs about the risks and benefits of a specific behavior) produce an attitude toward the behavior. Normative beliefs about the behavior (ie, consumers’ beliefs about how other people would like them to perform) will generate subjective norms. Finally, control beliefs (ie, consumers' beliefs about the facilitators or barriers that influence their ability to control the behavior) will generate the perceived behavioral control in relation to the behavior [23]. The TPB was employed in this study as a conceptual framework to develop the coding of themes.

## Methods

### Data Collection

Data were collected using ProQuest, one of the largest newspaper databases on the internet. The objective was to retrieve articles covering the purchasing of fake prescription medicines on the internet by members of the public. By a consensus among the authors (HA, NP, and PD), the search was carried out using the following search strategy: *(SU.EXACT(“pharmaceutical product”) OR SU.EXACT(“pharmaceutical products”) OR medicine OR medicines OR drug OR drugs OR medication OR medications OR remedy OR remedies) AND (On the internet OR internet OR web OR net) AND (Fake OR falsified OR counterfeited OR substandard)*. The search was limited to full-text news articles published in English between April 2019 and March 2022. The following databases were searched: International Newsstream‎, Global Breaking Newswires, US Newsstream‎, and Canadian Newsstream.

**Figure 2:**
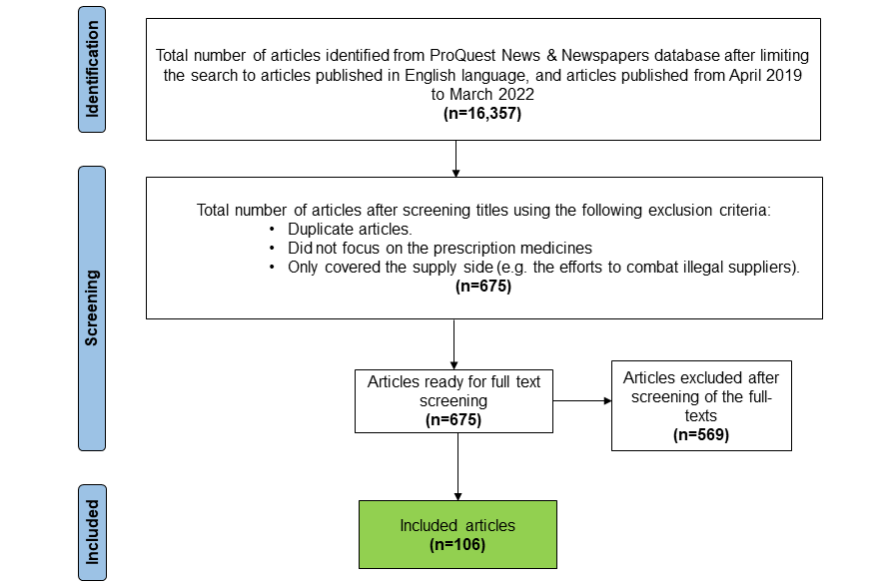
Flow diagram of the selection process.

No article was excluded based on the country of origin or source so that we could obtain a diversified view of the research topic. Figure 2 illustrates the flow diagram of the selection process. The searches revealed 16,357 articles, from which duplicates were removed; these were articles with the same content published in different versions of a newspaper (eg, Sunday and weekday editions) and articles that appeared in both print and web versions of the same newspaper (eg, appearing in both *The Telegraph* and telegraph.co.uk). Additionally, articles that did not focus on prescription medicines and those that only covered the supply side of fake medicines (eg, efforts to combat illegal sellers of fake medicines) were excluded. The articles yielded were then reviewed by the first author (HA), who screened the titles and content of each article and collected all potentially ambiguous articles for discussion with the second and third authors (NP and PD). After evaluation of the articles using the exclusion criteria, 106 articles remained and constituted the study sample. Multimedia Appendix 1 shows the characteristics of the selected articles (ie, titles, publication date, publishers, and locations).

### Data Analysis

Our aim was to analyze the retrieved articles to deconstruct and then reconstruct data to explore the newspaper coverage of the problem of purchasing fake prescription medicines on the internet. To do this, thematic analysis was employed according to the procedure set out by Braun and Clarke [24]. This process was carried out by the first author (HA) and reviewed step-by-step by the other authors (PD and NP). In summary, newspaper articles were read and reread in depth before being coded. Then, the articles were coded and organized into initial thematic groups (themes). The articles were then analyzed and organized using NVivo software (version 12; QSR International). Finally, we reviewed and gathered the initial themes into overarching themes (superordinate themes) in an iterative process guided by our study aim.

### Ethics Approval

No ethical approval was required as all the newspapers analyzed are available in the public domain.

## Results

### Overview

A total of 106 articles met the inclusion criteria and were included in the analysis (Multimedia Appendix 1). Regarding the distribution of articles based on the location, the majority of articles were published in the United Kingdom (n=47, 44.3%), followed by the United States (n=26, 24.5%), Asia (n=17, 16.1%), Canada (n=5, 4.7%), Australia (n=4, 3.8%), Africa (n=4, 3.8%), and Ireland (n=3, 2.8%).

**Figure 3:**
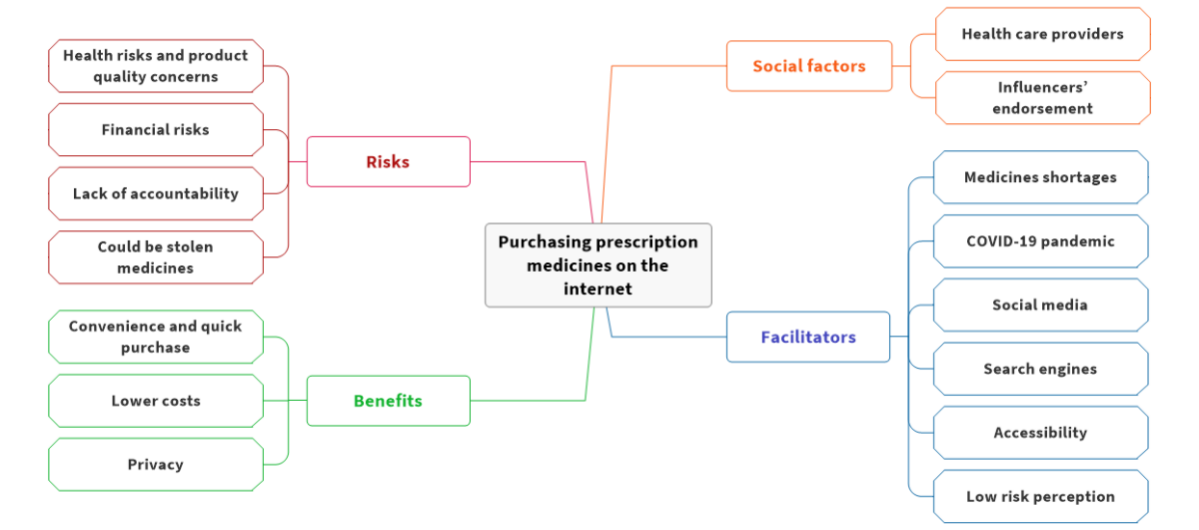
Analysis mind map.

The thematic analysis of the included articles revealed four superordinate themes: (1) the risks of purchasing prescription medicines on the internet, (2) the benefits that entice consumers to purchase prescription medicines on the internet, (3) social influencing factors, and (4) the facilitators of purchasing prescription medicines on the internet. A detailed mind map is shown in Figure 3.

### Risks of Purchasing Medicines on the Internet (Behavioral Beliefs)

The first superordinate theme to appear presented the risks associated with purchasing prescription medicines on the internet (Table 1).

There was a belief by part of the public that medicines offered on the internet possess a health risk due to the absence of medical oversight, as patients could engage in self-medication, which increases the possibility of misusing or abusing medicines. An additional serious risk highlighted was the risk of fake medicines being available on the internet. For example:

Some medicines bought online have been found to contain dangerous toxic substances. Because there is no certainty about what is in the medicine you buy on the internet, you can't be sure if it is safe to use alongside medicines you may already be taking. There could be interactions with your regular medicines.

Philadelphia Tribune, December 22, 2020; article 65 in Multimedia Appendix 1

Another risk highlighted by the news articles was the possibility of purchasing low-quality medicines, as these might be ineffective due to an absence of active ingredients or an incorrect quantity of these ingredients. Moreover, medicines available on the internet might not meet the standards of quality and hygiene, might not have been stored correctly, and could even contain expired ingredients. For example:

There is no way to be certain how or where the medicine was made. This means you cannot know if the manufacturer operates to acceptable standards of quality and hygiene. The medicine may not have been packaged, labelled or stored correctly and could be out-of-date. The active ingredient in a medicine is what makes it work. A medicine bought online may contain no active ingredient, too much or too little of an active ingredient or the wrong ingredients altogether. It could be useless in treating your medical complaint.

Philadelphia Tribune, December 22, 2020; article 65 in Multimedia Appendix 1

**Table 1:** Risks of purchasing prescription medicines on the internet.

Theme	Code
Health risks and product quality concerns	Lack of medical oversight Risks of fake medicines Medicines could be ineffective (expired medicines, inappropriate storage conditions)
Financial risks	Financial losses Identity theft
Lack of accountability	Lack of accountability
Risk of purchasing stolen medicines	Medicines sold on the internet could be stolen

The financial risks associated with these internet purchases (including the risks of incurring financial losses or the risk of identity thieves who could illegally access personal information) were pointed out in some news articles. For example:

…There are reports of fraudsters falsely claiming to be from legitimate health organizations, selling fake medicines, vaccines, tests, medical supplies, and using phishing scams to steal personal and financial information. Please don't fall victim to these frauds and crimes.

Targeted News Service, March 25, 2020; article 35 in Multimedia Appendix 1

The lack of accountability was highlighted as another risk of buying prescription medicines on the internet, where no one will be accountable if any problems occur with the purchase:

Rogue Internet registrars that knowingly facilitate illegal online pharmacies epitomize the types of notorious markets that USTR admirably seeks to combat […] ASOP Global urges USTR to support specific, no-cost and common-sense policy measures to hold offenders accountable […] Momentum is building for structural and policy reforms to increase accountability for internet ecosystem actors-there are downstream effects on the global illicit drug trade.

Targeted News Service, February 1, 2020; article 85 in Multimedia Appendix 1

One other risk highlighted was that the medicines available on the internet could have been stolen:

South Africa's statutory health regulatory bodies have sent out a red alert fearing that medication stolen during last week's looting could be consumed by people without the supervision and guidance of health-care professionals […] SAHPRA has warned people not to buy medicines online or from unknown sources.

The Daily News, July 20, 2021; article 98 in Multimedia Appendix 1

### Benefits That Entice Consumers to Purchase Prescription Medicines on the Internet (Behavioral Beliefs)

Another theme that appeared was the benefits that consumers could experience if they decide to purchase prescription medicines on the internet (Table 2).

For example, there was the belief that buying prescription medicines on the internet is more convenient because medicines can be purchased easily and delivered promptly to the consumer’s home:

I bought them online. They’re easy to get, and they’ll change everything. I logged on to Day Night Healthcare, an online pharmacy based in India, and ordered a pack of abortion pills […] and in a week and a half, a small brown envelope —bearing a postmark not from India but from New Jersey —arrived in the mail.

The New York Times, August 5, 2019; article 3 in Multimedia Appendix 1

There was also the belief that purchasing prescription medicines on the internet could save time and effort by avoiding long waiting times to book an appointment with a doctor, which could be over 2 years in some situations. In contrast, if consumers select the internet route, they can obtain their medicines within days to weeks. The following extract illustrates this point from the perspective of a transgender woman who would otherwise face a long wait for the hormonal medicine they wanted:

Law student Kara told the BBC's Victoria Derbyshire programme she began buying oestrogen - the hormone prescribed to trans women-last summer online, to begin her transition. She has been on an NHS gender identity clinic waiting list now for two years […] She told the BBC she felt the waiting times had “forced” her to make the choice to buy hormones online.

BBC News, February 18, 2020; article 68 in Multimedia Appendix 1

**Table 2:** Benefits enticing consumers to purchase prescription medicines on the internet.

Theme	Code
Convenience and quick purchase	Easy to obtain Fast delivery Saves time by avoiding long waiting lists to be treated by doctors
Lower cost	Cheaper prices More affordable for medicines that are not covered by insurance or that require a high surcharge
Privacy	Avoid admitting illness or health condition (eg, addiction, sexual dysfunction, overweight)

Another benefit highlighted by the newspaper articles was the lower cost to consumers. Some articles discussed how the low prices of medicines available on the internet could entice consumers to source their medicine on the internet, which is particularly helpful for patients or medicine not covered by medical insurance. The following 2 examples illustrate this point:

Unlicensed versions bought online and from abroad can cause breathing problems, experts warn. Prof Mahendra Patel, of Bradford University, said: “People have been hospitalised. Sometimes people are led by price. Your health should be the priority.”

The Daily Mirror, August 30, 2019; article 9 in Multimedia Appendix 1

Faced with soaring costs and insurance restrictions, Minnesota diabetics are turning to Facebook, eBay, Craigslist, and other lesser-known markets where they can offer medication they no longer need and ask others for help. Reselling a prescription medication such as insulin, or even giving it away for free, is illegal under federal and state laws.

Star Tribune, June 24, 2019; article 12 in Multimedia Appendix 1

The privacy of an internet purchase was highlighted as a factor that averts the need to consult a doctor or obtain a prescription. This is especially the case for sensitive health issues, where the shame of admitting a condition acts as a barrier to seeking care (eg, disclosing sexual dysfunction, addiction to psychoactive medicines, or using slimming pills for weight loss). The following newspaper excerpt highlights this:

Why does, as a British study reports, a young man prefers buying potency medicines such as Viagra at an obviously illegal internet pharmacy instead of going to a high street pharmacy? And what leads a young woman to act in a similar way to get slimming pills rather than go to a doctor? Clearly, this could be down to people feeling ashamed to openly admit sexual dysfunction or problems with losing weight.

iNews, June 27, 2019; article 26 in Multimedia Appendix 1

### Social Influencing Factors (Social Beliefs)

This superordinate theme focuses on the social factors that influence the purchase of prescription medicines on the internet (Table 3).

**Table 3:** Social influencing factors.

Theme	Code
Influencers	Endorsement by influencers such as politicians
Health care providers	Health care providers like doctors and pharmacists

Influencers, including politicians, who have a large number of followers were highlighted as a factor that could influence people to buy prescription medicines on the internet. For instance:

Online demand for the anti-malaria drug hydroxychloroquine surged by more than 1,000% after Donald Trump endorsed it as a potential treatment for COVID-19 without providing evidence it worked, a new study has found.

The Guardian Online, April 29, 2020; article 40 in Multimedia Appendix 1

Health care providers were highlighted as influencing people’s decision to buy prescription medicine on the internet by educating people on how to buy the medicines safely, as exemplified in the following extract:

The survey's findings underscore the critical role that healthcare providers play in educating consumers about how to buy medicines online safely

PR Newswire, October 19, 2020; article 53 in Multimedia Appendix 1

### Facilitators of Purchasing Prescription Medicines on the Internet (Control Beliefs)

Several facilitators and facilitating conditions that can trigger consumers to purchase medicines via the internet were highlighted in the newspaper articles (Table 4).

**Table 4:** Facilitators of purchasing prescription medicines on the internet.

Theme	Code
Medicine shortages	Medicines shortages, which might be caused by a halt in production, increased demand, lack of alternatives, or political events (eg, Brexit)
Pandemic disease (COVID-19)	Panic buying of medicines due to the fear of medicines running out of stock or being in shortage On the internet availability of COVID-19 cures (fake cures) for people desperate for treatment for COVID-19 Spread of misinformation related to COVID-19 Overburdened health care services
Social media	Facilitate communication between buyers and sellers
Search engines	Facilitate marketing of web-based sellers of medicines and thus help people find those sellers
Accessibility	People can purchase prescription medicines without the need for prescriptions
Low risk perception	Unawareness about the risks associated with purchasing medicine on the internet

One of the facilitating conditions was medicines shortages, which could be attributed to several reasons, such as halting the production of specific kinds of medicines or the increase in demand for specific medicines. It is also possible for a political event to affect the supply chain of medicines, such as fears about the impact of Brexit (ie, the United Kingdom leaving the European Union) on drug distribution and the supply chain. The following is an example extracted from a newspaper that covers the recent hormone replacement therapy shortages in the United Kingdom:

“Women are getting desperate, they are left with no other option than to look overseas,” she told the Daily Mail. The shortages have prompted fears that women are turning to buying potentially counterfeit HRT-drugs online.

Telegraph.co.uk, August 24, 2019; article 19 in Multimedia Appendix 1

Another facilitator highlighted by the newspaper articles was the outbreak of pandemic viruses, such as COVID-19. Illegal web-based pharmacies took advantage of the high market demand for medicines and personal protection products as well as panic buying (ie, purchasing prescription medicines on the internet from any source during the COVID-19 pandemic due to fears over prescription medicines running out of stock) to make mass profits from the sale of (fake) medicines:

The Maharashtra Cyber Police have busted an online fraud of illegal sales of fake Remdesivir and Tocilizumab drugs filled with plain water that targeted gullible customers desperate for treatment of Covid-19

IANS English, May 13, 2021; Article 94 in Multimedia Appendix 1

In addition, health services overburdened by COVID-19 meant that people struggled to get appointments with doctors and thus turned to the internet for treatment, including prescription medicines. For example:

A Daily Express investigation found powerful prescription-only cancer drugs readily available to UK citizens without prescription through an online pharmacy. The dangers are clear as millions of Britons, struggling to get NHS appointments, turn to the internet for cures, bypassing traditional and safer medical routes for diagnosis and treatments […] Interpol believes crime cartels are cashing in on the growing market because of the vast profits to be made from exploiting people's fears while health services are under pressure from covid.

Daily Express, August 20, 2021; article 86 in Multimedia Appendix 1

Social media platforms were pointed out by the newspaper articles as a facilitator for purchasing prescription medicines, as they offer marketplaces that allow for easy communication between consumers and illegal sellers of medicines. For example:

Cyber police issued an advisory on Friday warning citizens to not fall prey to conmen posing as pharma firm representatives on social media and offering to sell Remdesivir and Tocilizumab [...] “Con men are taking advantage to dupe those in need of the injections. No individual, distributor or retailer can sell these medicines to the public... beware of fraudsters... do not transfer funds and inform the police.

The Times of India, May 1, 2021; article 87 in Multimedia Appendix 1

Search engines such as Google were also highlighted by the newspaper articles as facilitators of purchasing prescription medicines via the internet. Search engines helped people find websites that offer prescription medicines without the need for prescriptions. For example:

Google is showing search results clearly marked as ”sponsored“ when certain keywords are used to search for the drug. The tech-giant is profiting by displaying these prominent shopping ads and, by extension, facilitating the [sale] of this potentially lethal drug.

Mail on Sunday, July 26, 2020; article 74 in Multimedia Appendix 1

The accessibility to prescription medicines without a need for a prescription was highlighted as another facilitating factor. For example:

The “skinny jabs,” which contain a prescription-only appetite suppressant known as Saxenda, are meant only for people with a BMI of over 30 or those with disorders such as diabetes. But many fake online pharmacies and unregistered clinics are now selling the jabs to people who are not overweight and do not have a prescription.

The Sun, February 23, 2020; article 2 in Multimedia Appendix 1

Finally, consumers’ low perception of the risks associated with purchasing medicines via the internet was discussed by the newspaper articles as another facilitating factor:

The survey findings demonstrate that American consumers believe they are more knowledgeable regarding online pharmacies than they are in reality. Further, most are unaware of the risks associated with their use or how to find legitimate sources versus rogue outlets, exposing millions more US consumers to the risk of potential fraud and criminality online.

PR Newswire, October 19, 2020; article 53 in Multimedia Appendix 1

## Discussion

### Principal Results

As more people are turning to the internet to purchase prescription medicines, newspaper coverage of this problem has also increased. This is the first study that analyzed newspaper coverage of the problem of purchasing fake prescription medicines on the internet. The analysis of the included articles revealed four major themes relating to purchasing prescription medicines on the internet, which include (1) the risks associated with the purchase, (2) the benefits that entice consumers to make the purchase, (3) the social influencing factors, and (4) factors that facilitate the purchase. Many of the analyzed articles included more than 1 theme.

Although this study explored newspapers in several countries, which have different health care systems as well as cultural differences among consumers, there was a consensus about the growing serious public risks and patient safety concerns associated with purchasing fake prescription medicines on the internet. Of course, this analysis reports on what newspapers covered rather than the truth of their coverage. In that sense, the articles are treated as data and taken at face value.

The newspaper articles highlighted that consumers were enticed by the convenience, low prices, and privacy afforded by purchasing prescription medicines on the internet. However, assurances of quality, efficacy, and safety cannot be guaranteed if the medicines are purchased via the internet from an illegal source. Moreover, if any unpleasant consequences occur (eg, financial scams, purchasing fake medicines), there is no accountability from the illegal sites. These findings are in line with those of several other studies [2,25-29].

The newspapers also highlighted several external facilitating conditions that could affect consumers’ decision to purchase prescription medicines from the internet, such as medicine shortages. Shortages of medicines caused by a political event (eg, Brexit) or supply chain issues can open the doors to sellers of prescription medicines on the internet. When there are limited sources available to obtain much-needed prescription medicines, this is likely to lead people to use internet sources, even if they are illegal and unsafe. Relating this to the previous literature, this finding is in line with that of a multinational study, which found that oncology medicines affected by shortages were available and accessible on the internet without the need for a medical prescription [30]. Furthermore, in an earlier study conducted in the United States that explored the internet availability of vaccines in shortages, it was suggested that vaccine shortages may lead some patients to seek out alternative sources (including the internet) even if they are not safe [31]. In line with this, a multinational study, which explored consumer behavior of purchasing lifestyle medicines from the internet, found that some consumers were willing to accept safety risks and buy prescription medicines on the internet during emergencies, such as medicine shortages [28].

Pandemics such as COVID-19 were highlighted by many newspaper articles as a facilitating condition that could trigger the panic buying of prescription medicines from any source available on the internet, mostly caused by the fear of prescription medicines running out of stock and feelings of desperation due to an absence of effective COVID-19 cures. Additionally, newspaper articles described how COVID-19 limited access to health care services due to overburdened hospitals and medical staff, which could drive some people to purchase prescription medicines on the internet. Interestingly, we found that many newspaper articles highlighted the impact of COVID-19 on the on internet purchases of prescription medicine; however, few studies have explored this issue [25,32]. Thus, further research might be needed to clarify the impact of COVID-19 on the purchasing of prescription medicines via the internet.

Another important facilitator highlighted by the newspaper articles was the easy accessibility of prescription medicines offered by illegal sellers without the need for a prescription. This result is in line with a study conducted in 2012 [33], which found that 55% (41/75) of the study sample preferred to purchase tramadol (a controlled prescription medicine) on the internet due to its easy access, especially when the doctor refused to prescribe this medication. Likewise, a recent study conducted in the United Kingdom [25] purported that the easy accessibility to prescription medicines on the internet without the need for a prescription can lead people to purchase these types of medications from the internet.

An interesting finding of this study was the ways in which the impact of social media on the purchase of prescription medicines via the internet was described in newspapers. Relating this to research findings, a multinational study found that pregnant women were influenced by support groups available on social media, which provided advice on how to use the medicines or where to source these medicines [34]. Another study conducted in Sweden uncovered many Facebook groups that sold illegal substances, including prescription medicines. The results of this study provide lend further support to the idea highlighted by the newspapers that social media can be a facilitator of communication between buyers and sellers that encourages the purchasing of prescription medicines on the internet [35].

This theory-based study also noted that purchasing fake prescription medicines on the internet can have implications for practice in terms of patient safety. Policy makers should take note of the potent power of news media as an influencer of the public. Newspaper articles are not only reporting and covering the topic of fake medicines available on the internet but can also influence people’s decisions and might help protect consumers from the risks of this purchase. To develop a full picture of this problem, additional studies are needed that explore and interpret this consumer behavior and provide behavioral change strategies that could deter people from purchasing prescription medicines on the internet.

### Limitations

The findings of this study are subject to some limitations. First, non-English language articles were excluded, which might affect the generalizability of our findings and increase the risk of bias. Another limitation relates to the sample of news sources selected for exploration. This study was limited to newspaper articles and internet news websites and thus excludes perspectives from other news media such as social media, television, and magazines.

### Conclusion

This study explored the news media coverage of the problem of purchasing prescription medicines on the internet by highlighting the risks associated with this purchase, the potential benefits that entice consumers to make this purchase, social influencing factors, and the facilitators of the purchase. Policy makers must consider the power of news media as an influencer of the public, as the news media could influence peoples’ decisions to purchase prescription medicines over the internet. Future research conducted in this area is needed to identify the factors that lead people to buy prescription medicines via the internet. This will aid the development of interventions to reduce the purchasing of prescription medicines from unsafe internet sources, thus protecting people from the health and safety risks of taking fake medicines.

## Appendix

Multimedia Appendix 1 Characteristics of the included newspaper articles.

## Abbreviations

TPB: Theory of Planned Behavior
WHO: World Health Organization

## Data Availability

The data sets generated during and/or analyzed during this study are available from the corresponding author upon reasonable request.
