# Supplementary material for: News Media Coverage of the Problem of Purchasing Fake Prescription Medicines on the Internet: Thematic Analysis
Source: JMIR Form Res. 2023 Mar 21;7:e45147. doi: 10.2196/45147 (PMC10131998; doi:10.2196/45147)
Supplement: Multimedia Appendix 1 [file formative_v7i1e45147_app1.docx]

**Included Newspaper Articles**

| **#** | **Title** | **Year** | **Publisher** | **Location** |
| --- | --- | --- | --- | --- |
| 1 | Online HRT health warning | 18-Aug-19 | The People | UK |
| 2 | ‘Miracle’ weight loss injections meant for the clinically obese are sold openly online without prescription | 23-Feb-20 | The Sun | UK |
| 3 | Abortion Pills Should Be Everywhere | 05-Aug-19 | The New York Times | USA |
| 4 | Covid-Vaccine Scams Spread, Preying on an Anxious Public | 24-Feb-21 | Wall Street Journal | USA |
| 5 | China also battles opioid problems | 01-Jan-20 | Telegraph Herald | USA |
| 6 | Pharmacy regulator sets safety rules for online drugs purchases | 16-Apr-19 | The Guardian (Online) | UK |
| 7 | Meghan’s war on diet pills scammers | 11-Aug-19 | Sunday Mirror | UK |
| 8 | Criminals cashing in on pandemic panic; Everything from counterfeit medical masks to fake treatments found online | 29-Apr-20 | Standard - Freeholder | Canada |
| 9 | FAKE VIAGRA SEIZURES JUST KEEP ON | 09-Aug-19 | The Daily Mirror | UK |
| 10 | It is illegal use of the Duchess's name and we will be taking action | 11-Aug-19 | Sunday Mirror | UK |
| 11 | Dangerous coronavirus 'vaccines' being sold on dark web | 01-May-20 | The Canberra Times | Australia |
| 12 | Diabetics resort to black market | 24-Jun-19 | Star Tribune | USA |
| 13 | Q&amp;A | 24-Aug-19 | Daily Mail | UK |
| 14 | Men warned not to use 'natural Viagra' lichen because it could be toxic | 30-Aug-19 | Daily Star (Online) | UK |
| 15 | Scots teens 'blind to risks of sedatives' | 01-Sep-19 | Sunday Times | UK |
| 16 | Illegal 'skinny jabs' openly sold online | 23-Feb-20 | Sunday Times | UK |
| 17 | Deadly drug sold as Xanax in Greenville, police warn Drug Enforcement Unit recovers pill presses in Greer apartment | 24-Feb-21 | The Greenville News | USA |
| 18 | VIAGRA FOOLS | 09-Apr-19 | Daily Star | UK |
| 19 | HRT drugs rationed amid UK shortage set to last for months | 24-Aug-19 | Telegraph | UK |
| 20 | Fake vaccines, 'cures' for sale on dark web supplies | 30-Apr-20 | The Age | Australia |
| 21 | Student pills alert | 06-Oct-19 | The Sun | UK |
| 22 | STROKES WARNING OVER FAKE VIAGRA | 23-Apr-19 | The Sun | UK |
| 23 | FDA warns Ontario holistic clinic to stop selling fake coronavirus cures | 10-Mar-20 | National Post (Online) | Canada |
| 24 | Scamwatch | 02-Mar-20 | Southern Highland News | Australia |
| 25 | Social media awash with fake remedies as snake-oil sellers flog harmful products | 29-Mar-20 | Sunday Age | Australia |
| 26 | The drugs don't work | 30-Jul-19 | iNews | UK |
| 27 | Alliance for Safe Online Pharmacies Issues Public Comment on Patent & Trademark Office Notice | 10-Jan-21 | Targeted News Service | USA |
| 28 | From counterfeit medical masks to bogus medical treatments, criminals cashing in on pandemic panic | 29-Apr-20 | Daily Gleaner | Canada |
| 29 | UK joins global fight against 'dangerous' fake medicines touted online | 20-Mar-20 | The Daily Telegraph | UK |
| 30 | How COVID-19 fuels spike in fake drugs | 27-Aug-20 | The Guardian, Lagos | USA |
| 31 | INTERPOL Global Operation Sees a Rise in Fake Medical Products Related to COVID-19 | 20-Mar-20 | Targeted News Service | USA |
| 32 | Coronavirus scams infecting the Internet | 27-Feb-20 | Boston Globe | USA |
| 33 | 'I could get 500 tablets a month' the ease of | 26-Apr-19 | The Guardian (Online) | UK |
| 34 | Criminals pose as health officials to trick virus victims | 19-Mar-20 | Daily Mail | UK |
| 35 | Fraudsters cash in on coronavirus | 26-Mar-20 | Mmegi ; Gaborone | USA |
| 36 | Interpol warns of online scams offering fake Covid-19 vaccines, treatments | 09-Dec-20 | Indian Express | India |
| 37 | It only takes one ... there is no second chance | 20-Aug-20 | News - Star | USA |
| 38 | U.S. Attorney U.S. Attorney COVID-19 Fraud | 25-Mar-20 | Targeted News Service | USA |
| 39 | Deadly dangers of mail order medicine | 27-Oct-19 | Sunday Mirror | UK |
| 40 | Online demand for hydroxychloroquine surged 1,000% after Trump backed it, study finds | 29-Apr-20 | The Guardian (Online) | UK |
| 41 | Fake abortion pills targeting Maltese women on Facebook, activists warn | 16-May-20 | MaltaToday | USA |
| 42 | Public warned not to buy medicine online which claims to cure Covid-19 | 18-Sep-20 | Irish Examiner | Ireland |
| 43 | Online markets deal in death | 17-Jul-19 | The Times | UK |
| 44 | 'Stop Sales by Unlicensed E-pharmacies' [Companies Pursuit of Profit] DCGI asks state drug regulators to implement Delhi HC order that barred entities without licences from selling medicines online | 04-Dec-19 | The Economic Times (Online) | India |
| 45 | ILLICIT MEDICINES SEIZED IN SWOOPS | 20-Mar-20 | The Daily Mirror | UK |
| 46 | UK online pharmacies accused of 'aggressive' tactics to sell opiates | 26-Apr-19 | The Guardian | UK |
| 47 | Dangerous fake drugs bought online with Google | 09-Sep-19 | Sunday Times | UK |
| 48 | Council of Europe COVID-19 - How to Protect Against Falsified Medical Products | 09-Apr-20 | Targeted News Service | USA |
| 49 | Scammers Are Setting Up Fake Covid Vaccine Websites | 26-Feb-21 | Wall Street Journal (Online) | USA |
| 50 | Meghan Markle fury as scammers claim she used dangerous diet pills after Archie's birth | 11-Aug-19 | Daily Star (Online) | UK |
| 51 | Online 'miracle cures' pose risk to health, warns | 04-Apr-20 | Telegraph.co.uk | UK |
| 52 | Online pharmacy Chemists, druggists to meet min today | 28-Aug-19 | The Times of India | India |
| 53 | New Survey Reveals Dangerous Disconnect in American Perceptions of Online Pharmacies More Consumers Buy Medicine Online Despite Not Knowing the Risks of Illegal Internet Drug Sellers | 19-Oct-20 | PR Newswire | USA |
| 54 | Public warned against buying illicit medicines | 27-Sep-19 | The Mercury | South Africa |
| 55 | Over 2,500 online listings of health products removed Items made false, misleading claims or were adulterated Health Sciences Authority | 20-Mar-20 | The Straits Times | Singapore |
| 56 | Shopping abroad for drugs How to avoid legal risks and bad medication | 11-Sep-19 | Chicago Tribune | USA |
| 57 | Pharmaceutical association urges DCGI to stop online pharmacies | 17-Aug-19 | Daily News & Analysis | India |
| 58 | National Association of Boards of Pharmacy | 06-May-20 | Targeted News Service | USA |
| 59 | Z-pills UK | 07-Mar-21 | The Sun | UK |
| 60 | Cyber crooks play on corona pandemic panic | 28-Apr-20 | The Times of India | India |
| 61 | Amanda Holden ‘furious after being falsely linked to diet pills’ | 01-Aug-19 | Daily Star (Online) | UK |
| 62 | AWARENESS CAMPAIGN ON RISKS OF BUYING MEDICINES ONLINE | 02-Oct-19 | Malaysian National News Agency | Malaysia |
| 63 | Prescription Drugs to Your Door, Nearly as Easy as Ordering Pizza | 03-Apr-19 | New York Times | USA |
| 64 | Struggling to perform Get a diabetes check | 31-May-20 | Mail on Sunday | UK |
| 65 | Be careful when considering buying any medication online | 22-Dec-20 | Philadelphia Tribune | USA |
| 66 | Chinese actresses jailed for selling fake diet pills | 20-Apr-19 | South China Morning Post | China |
| 67 | DOH warns public against buying medicines at online shopping sites | 04-Dec-19 | Manila Bulletin | India |
| 68 | Transgender patients self-medicating over NHS waits | 18-Feb-20 | BBC news | UK |
| 69 | Six arrested following seizure of prescription-only and unlicensed medicines in the West Midlands | 10-Mar-21 | M2 Presswire | UK |
| 70 | Seizures of illegal diazepam tablets double in a year - BBC News | 05-Dec-19 | BBC news | UK |
| 71 | UK joins global fight against 'dangerous' fake medicines touted online IN BRIEF | 20-Mar-20 | The Daily Telegraph | UK |
| 72 | Derry is awash with fake drugs. Too many young people are dying' | 06-Jul-19 | Derry Journal | UK |
| 73 | Don’t let our young fall prey to dangerous fake | 26-Jul-20 | The Independent (Daily Edition) | UK |
| 74 | Uncomfortable truth of online marketplaces | 26-Jul-20 | Mail on Sunday | UK |
| 75 | Chemists demand ban on online sale of medicines [Aurangabad] | 19-Dec-19 | The Times of India | India |
| 76 | Covid-19 spawns illegal trade of PPE and antiviral drugs | 01-Jul-20 | Mint | India |
| 77 | GPs may hand out 'benzos' in effort to save lives | 07-Mar-21 | Sunday Times | UK |
| 78 | PILLS, THRILLS &amp; BELLYACHES | 23-Apr-19 | The Sun | UK |
| 79 | Philippines: Cordillera FDA heightens drive vs. fake medicines | 10-Jul-19 | Asia News Monitor | Thailand |
| 80 | 'Seeing Others Suffer is Too Stressful' - Why | 07-Sep-19 | Targeted News Service | USA |
| 81 | UK medicines and medical devices regulator investigating 14 cases of fake or unlicensed COVID-19 medical products | 06-Apr-20 | Presswire | UK |
| 82 | WEB MEDS ALERT [Eire Region] | 28-Mar-20 | The Sun | UK |
| 83 | Cheaper drug prices in the U.S. shouldn't involve raiding Canada's medicine cabinet Importing prescription medication from us is a ridiculously simplistic idea that is untenable both politically and practically | 29-Jul-19 | The Globe and Mail (Online) | Canada |
| 84 | Five arrested following seizure of prescription-only and unlicensed medicines in the West Midlands | 11-Mar-21 | M2 Presswire | UK |
| 85 | Alliance for Safe Online Pharmacies Issues Public Comment on U.S. Trade Representative Notice | 01-Feb-21 | Targeted News Service | USA |
| 86 | NIL BY MOUTH... THE FAKE MEDICINES PUTTING LIVES AT RISK | 20-Aug- 2021 | Daily Express | UK |
| 87 | Beware of fake Remdesivir offer on social media | 01-May -2021 | The Times of India | India |
| 88 | 'Potentially very dangerous' NHS prescription scam emerges sparking serious warning | 21-Jan-2022 | Express (Online) | UK |
| 89 | Amazon reviews push horse dewormer for covid, despite FDA warnings | 03-Sep-2021 | The Washington Post | USA |
| 90 | Seizure of illegal medicines up 58% in past year | 09-Jun- 2021 | Irish Times | Ireland |
| 91 | Alert over drugs claiming to cure cancer and autism | 11-Jan- 2022 | Daily Mail | UK |
| 92 | Louisiana Illuminator - States Newsroom: Scam online pharmacies selling fake, dangerous pills find opportunity in the pandemic | 12- Oct- 2021 | Weblog post | USA |
| 93 | Counterfeit prescription drugs | 25-Mar- 2022 | The Deming Headlight | USA |
| 94 | Maha Cyber cops bust racket of 'water-filled' Remdesivir, Tocilizumab drugs | 13-May-2021 | IANS English | India |
| 95 | US drug overdoses top 100,000 in pandemic year | 18 Nov 2021 | Manila Bulletin | India |
| 96 | FDA warns public vs fake drug products being sold online | 04 Oct 2021 | Manila Bulletin | India |
| 97 | Spike in prescription and fake drug misuse | 25-Jun- 2021 | Derry Journal | UK |
| 98 | Warning against using, selling stolen medication | 20 July 2021 | The Daily News | South Africa |
| 99 | Public urged not to buy Covid-19 drugs online after seizures | 14 Sep 2021 | Irish Times | Ireland |
| 100 | 'I felt an overwhelming panic' Woman, 48, devastated after losing £148 in online scam | 25-Feb-2022 | Express | UK |
| 101 | Online pharmacies can be risky | 31 Oct 2021 | The Jackson Sun | USA |
| 102 | Advisory - Be informed: know the potential risks of buying health products online | 14- Jun- 2021 | The Bassano Times | Canada |
| 103 | NAFDAC, FCCPC Helpless As Unregistered Drugs Take Over Internet Markets | 23 Jan 2022 | The Daily Trust | Nigeria |
| 104 | Do fake Covid-19 vaccine arrests in China herald global crime wave? | 23- May - 2021 | South China Morning Post (Online) | China |
| 105 | Ivermectin use could be harmful | 08-Sep-2021 | Durban | South Africa |
| 106 | ‘Dangerous!’ NHS free prescription warning as Britons trying to ‘save money’ at risk | 30-Jan-2022 | Express (Online) | UK |
